# A Champion of Host Defense: A Generic Large-Scale Cause for Platelet Dysfunction and Depletion in Infection

**DOI:** 10.1055/s-0040-1708827

**Published:** 2020-04-12

**Authors:** Martin J. Page, Etheresia Pretorius

**Affiliations:** 1 Department of Physiological Sciences, Stellenbosch University, Stellenbosch, South Africa

**Keywords:** platelets, virus, bacteria, thrombocytopenia, immune response

## Abstract

Thrombocytopenia is commonly associated with sepsis and infections, which in turn are characterized by a profound immune reaction to the invading pathogen. Platelets are one of the cellular entities that exert considerable immune, antibacterial, and antiviral actions, and are therefore active participants in the host response. Platelets are sensitive to surrounding inflammatory stimuli and contribute to the immune response by multiple mechanisms, including endowing the endothelium with a proinflammatory phenotype, enhancing and amplifying leukocyte recruitment and inflammation, promoting the effector functions of immune cells, and ensuring an optimal adaptive immune response. During infection, pathogens and their products influence the platelet response and can even be toxic. However, platelets are able to sense and engage bacteria and viruses to assist in their removal and destruction. Platelets greatly contribute to host defense by multiple mechanisms, including forming immune complexes and aggregates, shedding their granular content, and internalizing pathogens and subsequently being marked for removal. These processes, and the nature of platelet function in general, cause the platelet to be irreversibly consumed in the execution of its duty. An exaggerated systemic inflammatory response to infection can drive platelet dysfunction, where platelets are inappropriately activated and face immunological destruction. While thrombocytopenia may arise by condition-specific mechanisms that cause an imbalance between platelet production and removal, this review evaluates a generic large-scale mechanism for platelet depletion as a repercussion of its involvement at the nexus of responses to infection.

Infections, both bacterial and viral, are associated with a profound immune response to the infecting pathogen. Platelets are important contributors to the multifaceted response to infection, where they have the ability to modulate various immune cells. Platelets engage the immune system through direct cell-to-cell interaction and through the release of various soluble mediators. 1 2 3 4 5 Furthermore, platelets participate in the interaction between pathogens and host defense. 6 7 8 9 10 11 12 In the absence of platelets, bacteremia, tissue damage, and mortality are greatly enhanced. 13 14 15 Similarly, thrombocytopenia is associated with a dysregulated host response and worse outcomes in sepsis patients. 16 17 Platelets are also active participants in the host response to viruses, and have been shown to be protective in viral infections. 18 19 20

Platelets possess receptors that allow them to survey for danger signals from pathogens (pathogen-associated molecular patterns; PAMPs) and cell damage (damage-associated molecular patterns; DAMPs), and trigger hemostatic and inflammatory responses against bacterial and viral infections. 3 21 22 During infection, the platelet is activated, mobilized, and actively participates in the resultant hemostatic and inflammatory responses. These signaling processes involve many feedback loops that self-amplify initial activation, 23 and platelets can manifest dysfunction even in cases where no bacteremia is present. 10 These processes are irreversible and undoubtedly lead to consumption of the platelet. Activation of platelets leads to their consumption into aggregates with other platelets, leukocytes, and the endothelium. 24 Platelets with bound antibody are targets of phagocytes, and platelets with a bacterial or viral load are sequestrated and also cleared from the circulation. Further, pathogenic compounds induce apoptosis and cytotoxic effects in platelets. 25 In this sense, activated platelets and platelets interacting with pathogens have shortened survival spans and experience increased destruction. The outcome for the patient will be a decrease in normal circulating platelets, and if this manifests widely enough it can be measured as thrombocytopenia. 3 25

Other mechanisms of platelet decline in infection exist and include the formation of autoantibodies against platelet surface proteins, which leads to clearance of immunoglobulin G (IgG)-coated platelets by the reticuloendothelial system, 26 27 as well as by impaired platelet production in the bone marrow, 3 6 among others. 6 However, a general view of platelet destruction is the simple characteristic that their involvement in thrombotic, hemostatic, immune, and host defense responses is irreversible. Even if platelets are positive contributors to the host response against invading pathogens, they can become dysfunctional, especially in the context of an excessive and unbalanced systemic inflammatory response. 16 28 Indeed, the dysfunctional state of thrombocytopenia is commonly associated with sepsis and infections. 3 29 30 31

The focus of the current review is platelets and their role in infection. We will examine the interaction of platelets, their receptors, and secretory product with bacteria and viruses, and discuss how this may contribute to platelet dysfunction and ultimately lead to thrombocytopenia. Fig. 1 provides the rationale of this review and Table 1 lists the abbreviations used in this article.

**Fig. 1:**
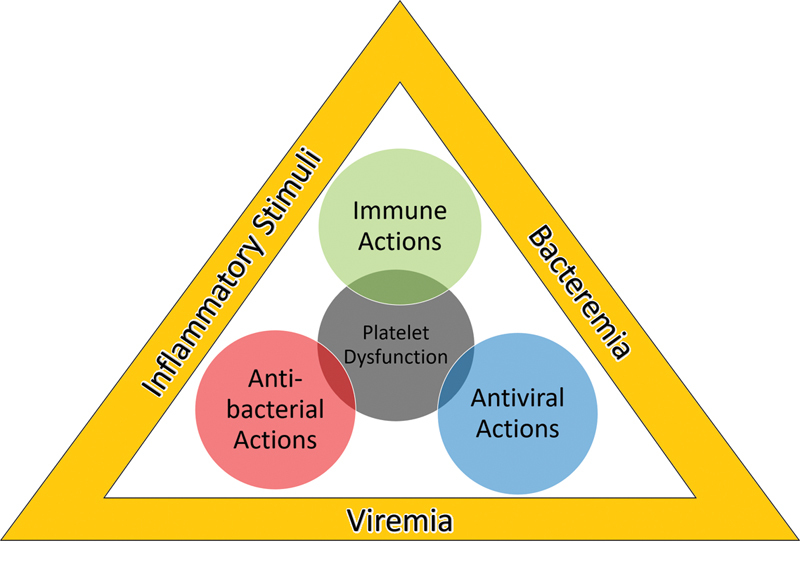
Layout of the review. During infection, inflammatory stimuli, and the presence of bacteria, viruses and their products mobilize platelets to exert their immune, antibacterial, and antiviral actions. However, these processes can also lead to platelet dysfunction and ultimately depletion.

**Table 1:** List of abbreviations

Abbreviation	Full term	Synonyms
αIIbβ3		GPIIb/IIIa
αMβ2	Macrophage-1 antigen	CD11b/CD18, CR3; Mac-1
cAMP	Cyclic adenosine monophosphate	
CAR receptor	Coxsackievirus and adenovirus receptor	
(s)CD40L	(Soluble) CD40 ligand	CD154
cGMP	Cyclic guanosine monophosphate	
CR2	Complement receptor 2	CD21, C3dR
CR3	Complement receptor 3	αMβ2, CD11b/CD18, Mac-1
CR4	Complement receptor 4	αxβ2, CD11c/CD18
DAMP	Damage-associated molecular pattern	
DNA	Deoxyribonucleic acid	
Eap	Extracellular adherence protein	
Efb	Extracellular fibrinogen binding protein	
FcγRIIa	Low affinity immunoglobulin gamma Fc region receptor II-a	CD32
GPIb	Glycoprotein Ib	CD42
GPVI	Glycoprotein VI	
HIV	Human immunodeficiency virus	
HLA-DR	Human leukocyte antigen—DR isotype	
HRgpA	Recombinant gingipain R1 protease (high molecular mass form)	
Ig	Immunoglobulin	
IL	Interleukin	
LCMV	Lymphocytic choriomeningitis virus	
LPS	Lipopolysaccharide	
LTA	Lipoteichoic acid	
MyD88	Myeloid differentiation primary response 88	
NET	Neutrophil extracellular trap	
P-selectin		CD62P, GMP-140, PADGEM
PAF	Platelet-activating factor	
PAMP	Pathogen-associated molecular pattern	
PAR	Protease-activated receptor	
PF4	Platelet factor 4	CXCL4
PKG	cGMP-dependent protein kinase	
PSGL-1	P-selectin glycoprotein ligand-1	CD162
RANTES	Regulated on activation, normal T-cell expressed and secreted	CCL5
RgpB	Recombinant gingipain R2 protease	
RNA	Ribonucleic acid	
ROS	Reactive oxygen species	
SSL	Staphylococcal superantigen-like	
TLR	Toll-like receptor	
TNF	Tumor necrosis factor	
TREM-1(L)	Triggering receptor expressed on myeloid cells 1 (ligand)	CD354

## Platelet and the Immune Response to Infections

A common feature of many infections, both viral and bacterial, is a systemic inflammatory response that involves a dysregulated proinflammatory biomarker presence in the circulation. 3 5 32 These biomarkers may include cytokines (e.g., interleukins [ILs], tumor necrosis factor [TNF]-α, and interferons) but also molecules originating from bacteria and viruses themselves (e.g., proteases, ribonucleic acid [RNA], and membrane components like lipopolysaccharide [LPS], lipoteichoic acid [LTA], and viral glycoproteins). The presence of such circulating biomarkers has profound agonistic effects on platelets.

**Fig. 2:**
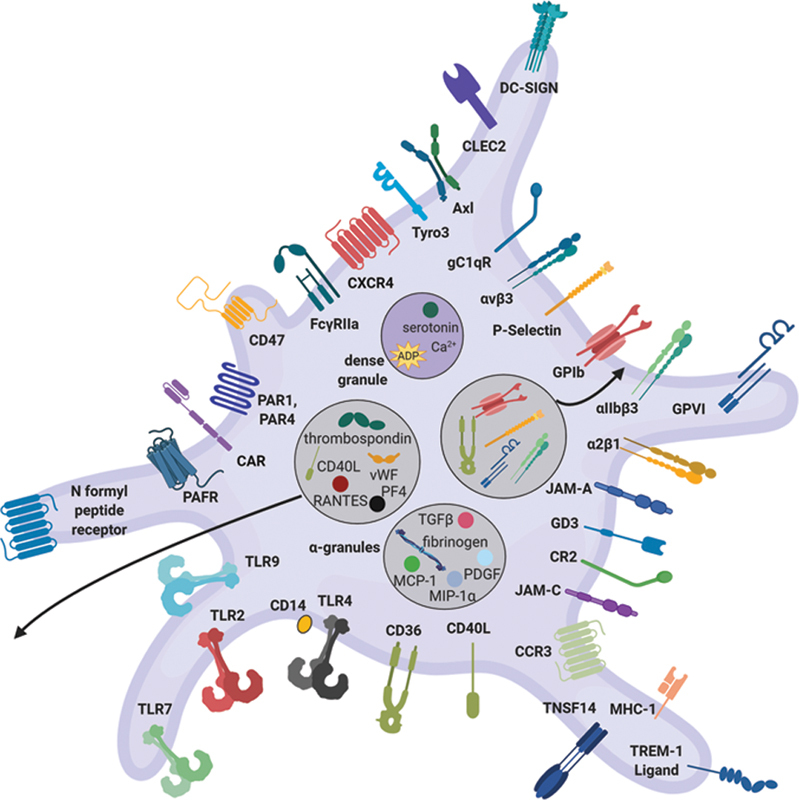
General platelet structure. Platelets express various receptors that allow them to detect danger signals and engage other cells. Platelets are activated by various agonists that interact with surface receptors. Platelets are also replete with secretory granules that store bioactive molecules, which are released into the circulation or translocate to the surface upon platelet activation. These characteristics allow platelets to communicate and modulate the functions of other cells, and trigger hemostatic, inflammatory, and host defense responses against infections (created with https://biorender.com/ ). ADP, adenosine diphosphate; CAR *,* coxsackievirus and adenovirus receptor; CCR/CXCR, chemokine receptor; CLEC, C-type lectin-like receptor *;* CR, complement receptor; DC-SIGN, dendritic cell-specific ICAM-grabbing nonintegrin; FcγRIIa, low-affinity immunoglobulin gamma Fc region receptor II-a; gC1Qr, receptor for the globular heads of C1q; JAM, junction adhesion molecule; MCP, monocyte chemoattractant protein; MHC, major histocompatibility complex; MIP, macrophage inflammatory protein; PAFR, platelet-activating factor receptor; PAR, protease-activated receptor; PDGF, platelet-derived growth factor; PF, platelet factor; RANTES, regulated on activation, normal T-cell expressed and secreted; TGF, transforming growth factor; TLR, toll-like receptor; TNSF14, tumor necrosis factor superfamily member 14; TREM, triggering receptor expressed on myeloid cells; vWF, von Willebrand factor.

Platelets contribute to the thromboinflammatory response through the plethora of membrane and cytosolic molecules that they express and release, which possess hemostatic, immunomodulatory, and inflammatory activity. 1 2 3 4 Platelets possess receptors that enable pathogen sensing, and which allow platelets to regulate leukocytes and other cells at the site of infection. During platelet activation, degranulation leads to the surface expression of receptors and the release of abundant proinflammatory mediators, which contribute to numerous signaling events. 1 2 3 4 5 Platelets also adhere and aggregate to other platelets and to endothelial cells, leukocytes, and erythrocytes. 5 9 24 This response is also characteristic during bacterial and viral infections, and can be induced by pathogens directly. 33 This section describes the role of platelets in the immune response. See Fig. 2 for a general overview of platelet receptors and secretory products.

### Platelet–Endothelium Interactions: Endowing a Proinflammatory Phenotype

**Fig. 3:**
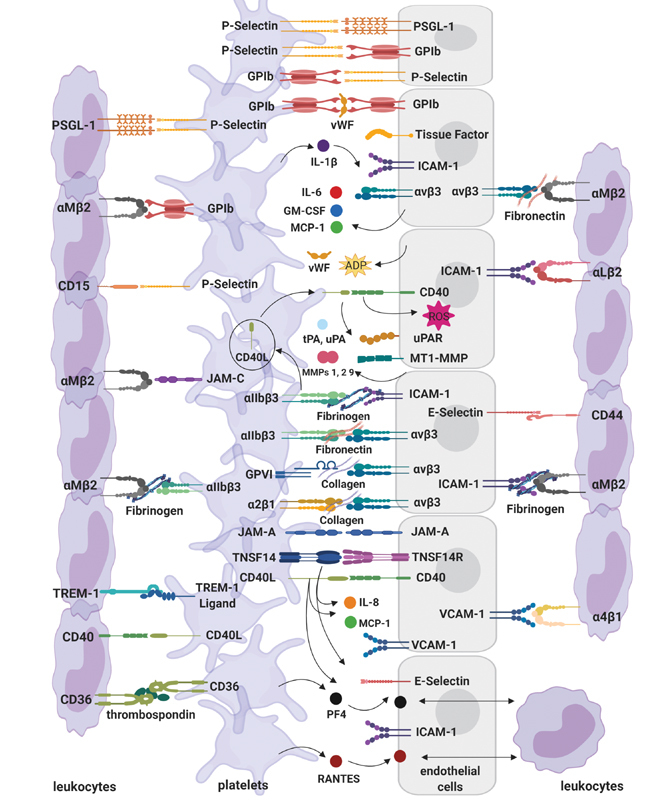
Platelet interactions at the vascular wall. Platelet activation and adhesion to the vascular wall is facilitated by various receptor interactions with endothelial cells. An inflamed vessel wall will adopt a prothrombotic phenotype and release platelet binding and stimulating agents. The adhesion of platelets activates endothelial cells, and together with potent inflammatory mediators released by platelets induces the expression of integrins, adhesion molecules, and other receptors on the endothelial surface, as well as causes the endothelium to secrete chemokines and other mediators. Platelets similarly bind and activate leukocytes, contributing to leukocyte recruitment to the endothelium. In turn, leukocytes are activated and are able to adhere to the inflamed vessel, with platelets also serving as bridging connections between the endothelium and circulating leukocytes (created with https://biorender.com/ ). (Adapted from van Gils et al 24 .) ADP, adenosine diphosphate; GM-CSF, granulocyte-macrophage colony-stimulating factor; ICAM, intercellular adhesion molecule; IL, interleukin; JAM, junction adhesion molecule; MCP, monocyte chemoattractant protein; MMP, matrix metalloproteinase; MTP1-MMP, membrane type-1 MMP; PF, platelet factor; PSGL, P-selectin glycoprotein ligand-1; RANTES, regulated on activation, normal T-cell expressed and secreted; ROS, reactive oxygen species; TNSF14(R), tumor necrosis factor superfamily member 14 (receptor); tPA, tissue plasminogen activator; TREM, triggering receptor expressed on myeloid cells; uPA, urokinase-type plasminogen activator; uPAR, urokinase receptor; VCAM, vascular cell adhesion protein; vWF, von Willebrand factor.

Endothelial activation markers are raised during infection, and are associated with a thrombotic state. 34 During activation, platelets can bind to the endothelium. 24 This especially occurs upon endothelial damage due to trauma or microbial colonization, 35 as well as in viral infections. 36 Platelets become activated during the adhesion process, and the inflammatory and mitogenic substances that are released alter the chemotactic, adhesive, and proteolytic properties of endothelial cells. 37 Platelet adhesion therefore endows the endothelium with a proinflammatory phenotype. 24 Moreover, platelets that are bound to the endothelium can form a bridging connection with circulating leukocytes. 24 Overall, these mechanisms amplify and facilitate leukocyte recruitment and enhance inflammation. Fig. 3 provides an overview of the contact between platelets and cells at the vascular wall to emphasize the involvement of platelets in multiple interactions at the vessel wall.

### Platelet–Leukocyte Interactions: Promoting Immune Cell Effector Functions against Pathogens

Interactions between platelets and leukocytes are important for the regulation of the immune response and for the clearance of infectious agents. By binding and activating leukocytes, platelets promote their effector functions. Coordination of immune cells by platelets ensures a rapid and targeted host defense response. In a dynamic cross-talk, leukocytes can also release factors that modulate platelet function.

Platelets adhere to phagocytes and deliver signals that enhance the killing of internalized pathogens. Platelets are able to modulate neutrophil responses where they enhance neutrophil phagocytosis in a process involving toll-like receptor (TLR) 2 and P-selectin/P-selectin glycoprotein ligand (PSGL)-1. 38 This was demonstrated for both *Aggregatibacter actinomycetemcomitans* and *Porphyromonas gingivalis* . 38 Platelets can augment the respiratory burst in neutrophils in response to opsonized *Escherichia coli* and *Staphylococcus aureus* . 39 Platelet–neutrophil complexes have more activated adhesion molecules, greater phagocytic ability, and greater toxic oxygen metabolites than noncomplexed neutrophils. 40 Activated platelets can also induce superoxide anion release by monocytes and neutrophils through P-selectin. 41 Soluble CD40 ligand (CD40L) further interacts with CD40 and αMβ2 on neutrophils to induce the adhesive functions of neutrophils as well as cause CD40-dependent reactive oxygen species (ROS) generation. 42

Additionally, the triggering receptor expressed on myeloid cells (TREM)-1 ligand is expressed on platelets and has been shown to induce neutrophil activation, and platelets enhance the neutrophil respiratory burst and release of IL-8 in a TREM-1-specific manner in the presence of LPS. 43 The TREM-1 receptor is an important receptor in the innate immune response as well as in severe sepsis where it amplifies the immune response to microbial products. 44 TREM-1 has also been shown to contribute to neutrophil activation in viral infections. 45

**Fig. 4:**
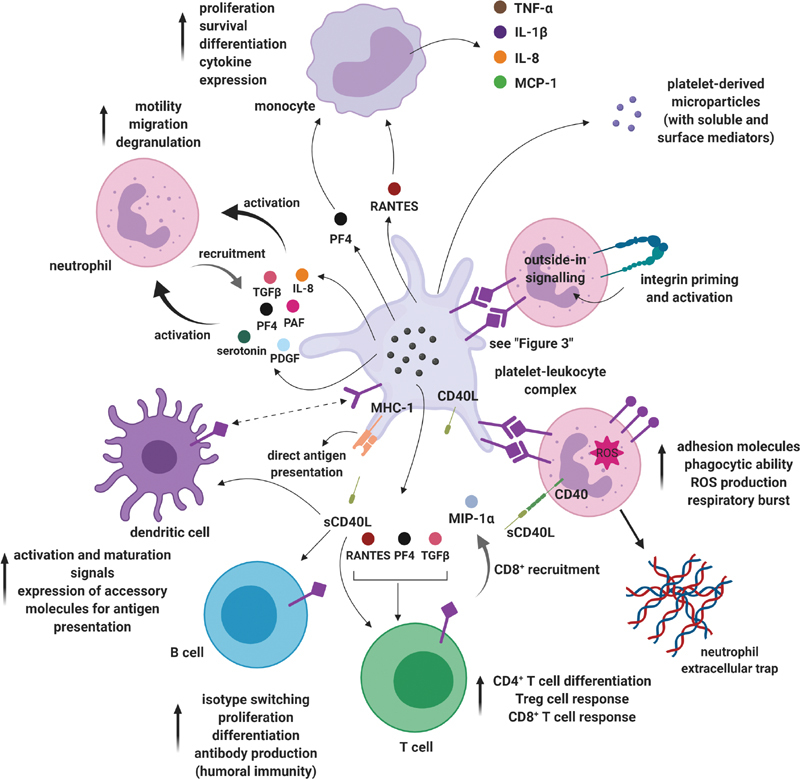
Platelet interactions with immune cells. Platelets are important contributors to the multifaceted immune response to infection and have the ability to engage the immune system. Degranulation leads to the surface expression of receptors and the release of abundant proinflammatory mediators that regulate leukocytes at the site of infection. Platelets also modulate leukocytes involved in adaptive immunity. Ultimately, platelets promote the effector functions of immune cells and enable an optimal immune response (created with https://biorender.com/ ). IL, interleukin; MCP, monocyte chemoattractant protein; MHC, major histocompatibility complex; MIP, macrophage inflammatory protein; PAF, platelet-activating factor; PDGF, platelet-derived growth factor; PF, platelet factor; RANTES, regulated on activation, normal T-cell expressed and secreted; ROS, reactive oxygen species; TGF, transforming growth factor; TNF, tumor necrosis factor; Treg, regulatory T cell.

Furthermore, platelets induce the release of neutrophil extracellular traps (NETs), deoxyribonucleic acid (DNA) covered with various antimicrobial nuclear and granule-derived molecules 46 that ensnare and kill pathogens, in response to bacterial (septic) stimuli. 39 47 48 This NET response has been documented in *E. coli* gram-negative sepsis and *S. aureus* gram-positive sepsis. 47 Platelets have further been shown to interact with neutrophils following viral challenge, leading to the release of NETs. 49 50 51 NETs also deliver antiviral factors such as myeloperoxidase 46 and α-defensin, 50 and capture viruses and promote their elimination. 51 Fig. 4 provides an overview of the interactions between platelets and immune cells to emphasize the involvement of platelets in the immune response.

### Platelet Involvement in Adaptive Immunity: Ensuring an Optimal Adaptive Response

Further to the innate immune response, platelets are also important for an optimal adaptive immune response. The periodontopathogens *A. actinomycetemcomitans* and *P. gingivalis* have been shown to induce expression of CD40L on human platelets via TLR2 and TLR4. 52 Platelets can modulate B and T cell responses to microbial pathogens through CD40L, and are able to induce isotype switching by B cells and augment CD8 ^+^ T cell function. 53 54 CD40L on platelets enable T cell priming and augment CD8 ^+^ T cell responses against bacterial pathogens by enhancing maturation signals to dendritic cells and lowering the threshold for cell activation 55 56 57 (compare with reports that platelets can have an inhibitory effect on dendritic cells 58 59 ).

Platelet-mediated modulation of the adaptive immune system has also been shown to enhance protection against viral re-challenge. 53 Platelets expressing integrin β3 and CD40L are essential for lymphocytic choriomeningitis virus (LCMV) clearance by virus-specific cytotoxic T cells, and protect the host from virus-induced interferon-α/β lethal hemorrhage. 18 Activated platelets can also contribute to immunopathology (e.g., liver damage) by accumulating virus-specific cytotoxic T cells at the site of inflammation in models of acute viral hepatitis. 60 Serotonin released from platelets is vasoactive and can further support viral persistence in the liver by reducing microcirculation, which aggravates virus-induced immunopathology in a model of LCMV-induced hepatitis. 61

Platelets can further shuttle blood-borne gram-positive bacteria to splenic CD8α ^+^ dendritic cells after the bacterium becomes associated to platelets via glycoprotein (GP)-Ib and complement C3 to balance bacterial clearance with immune induction. 62 Activated platelets also form aggregates with CD16 ^+^ inflammatory monocytes and human leukocyte antigen (HLA)-DR ^+^ CD38 ^+^ memory T cells in human immunodeficiency virus (HIV) infection. 7

### Platelet-Derived Microparticles: Further Driving the Inflammatory Response

Activated platelets produce microparticles during bacterial 63 64 and viral infection 65 66 that contain both soluble (e.g., regulated on activation, normal T cell expressed and secreted [RANTES]) and surface mediators (e.g., P-selectin, GPIb, and αIIbβ3), which can exit the vasculature and enter tissues where they are able to activate leukocytes to further drive the inflammatory response. 67 68 For example, platelet microparticles enhance the expression of cell adhesion molecules such as leukocyte αMβ2 for monocyte adhesion, 69 and can mediate leukocyte activation 70 and leukocyte–leukocyte interactions. 71 Microparticles promote platelet interaction with the endothelium by acting as a substrate for further platelet binding. 72 Further, microparticles can deliver platelet-derived CD40L signals 54 73 and activate dendritic cells. 74 Platelet microparticles also promote endothelial activation by secreting IL-1β, 75 and can deliver RANTES to the endothelium for monocyte recruitment. 76 Lastly, these microparticles can cause complement activation. 77

## Platelet Interactions with Bacteria

**Fig. 5:**
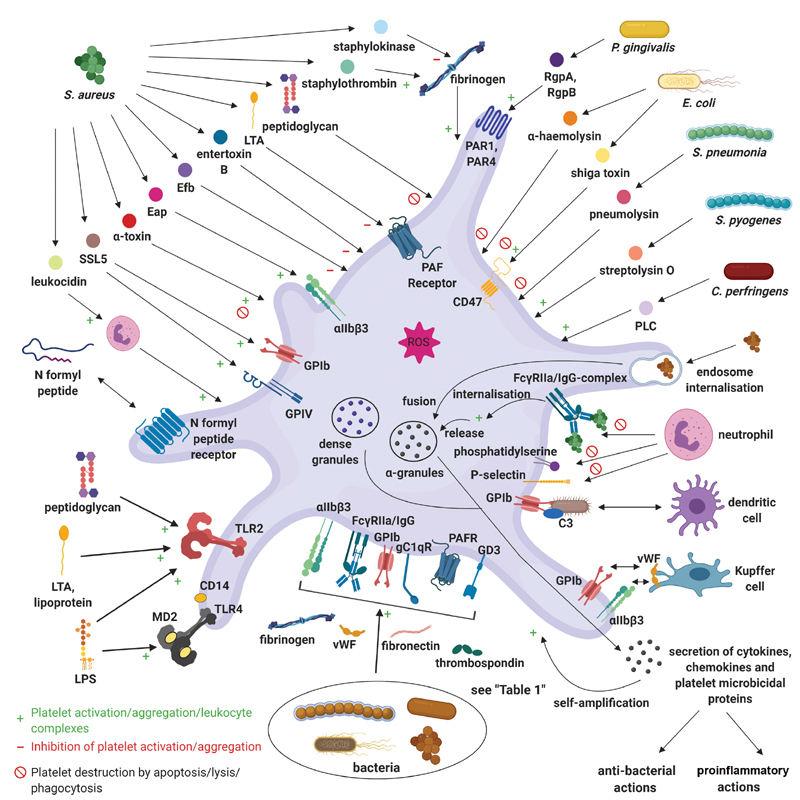
Platelet interactions with bacteria. Platelets are able to sense and bind bacteria through a variety of platelet receptors, and various bacterial products stimulate platelets, modulating their function. Platelets typically become activated and aggregate, but bacterial products may exert inhibitory actions or cause platelet destruction. Platelets additionally mediate antimicrobial actions by releasing microbicidal proteins, engulfing bacteria, and interacting with immune cells. These interactions further enhance the immune response and lead to platelet clearance (created with https://biorender.com/ ). C3 *,* complement component 3 *;* Eap, extracellular adherence protein; Efb, extracellular fibrinogen-binding protein; FcγRIIa, low-affinity immunoglobulin gamma Fc region receptor II-a; gC1Qr, receptor for the globular heads of C1q; Ig, immunoglobulin; LPS, lipopolysaccharide; LTA, lipoteichoic acid; PAF(R), platelet-activating factor (receptor); PAR, platelet-activating factor; PLC, phospholipase C; Rgp, recombinant gingipain; ROS, reactive oxygen species; SSL, staphylococcal superantigen-like; TLR, toll-like receptor; vWF, von Willebrand factor.

Platelets are active role players in antimicrobial defense, and exhibit complex interactions with bacteria and viruses due to the variety of platelet receptors involved in pathogen recognition. Platelets are able to recognize, bind, and internalize pathogens to sequester and neutralize the pathogen. This section describes the interactions of platelets with bacteria, which are summarized in Fig. 5 .

### Platelet Receptors in Bacterial Pathogen Sensing

It has long been known that bacteria can cause platelet aggregation and degranulation. 78 79 A diverse range of platelet receptors can mediate interactions with bacteria, including αIIbβ3, low-affinity immunoglobulin gamma Fc region receptor II-a (FcγRIIa), GPIb, complement receptors (CRs), and TLRs, 80 81 either directly or indirectly through bridging molecules. 11 12 81 Alternatively, products shed by bacteria 82 may cause a platelet response independently of direct bacterial attachment to the platelet. 10 Ultimately, engagement of receptors by bacteria and their products leads to common and species-specific intracellular signaling events in platelets. 83

Table 2 summarizes platelet receptors that mediate binding of bacteria to cause platelet activation and aggregation. A key mechanism for bacterial adhesion to platelets, which is described for various bacteria, involves αIIbβ3 integrin activation, the FcγRIIa receptor, and IgG, 84 where platelet factor (PF)-4 may potentiate further binding of additional bacteria by forming an immunocomplex with bacteria that bind through FcγRIIa. 85

**Table 2:** Platelet receptors that mediate bacterial adhesion and platelet activation

Bacteria	Bacterial component	Platelet receptors/host factors	References
*Borrelia burgdorferi*		αIIbβ3	182
*Chlamydia pneumoniae*		αIIbβ3	183
*Helicobacter pylori*		IgG-FcγRIIa, GPIb, vWF	184
*Porphyromonas gingivalis*	Hgp44	GPIb, IgG-FcγRIIa	185
*Streptococcus agalactiae*	FbsA	αIIbβ3, fibrinogen, IgG-FcγRIIa	186
*Staphylococcus aureus*	ClfA, ClfB, FnBPA, SdrE, SpA, IsdB	αIIbβ3, fibrinogen, fibronectin, IgG-FcγRIIa, complement gC1qR, thrombospondin, vWF	84 187 188 189 190 191 192 193 194 195 196 197 198 199 200 201
*Staphylococcus epidermidis*	SdrG	αIIbβ3, fibrinogen, IgG- FcγRIIa	202
*Streptococcus gordonii*	PadA, SspA/SspB, GspB/Hsa	αIIbβ3, GPIb, IgG-FcγRIIa	84 203 204 205 206
*Staphylococcus lugdunensis*	Fbl	Fibrinogen	207
*Streptococcus mitis*	PblA, PblB, lysin	αIIbβ3, fibrinogen, membrane ganglioside GD3	208 209
*Streptococcus oralis*		GPIb, IgG-FcγRIIa	84 210
*Streptococcus pneumoniae*	Pav, PspC/Hic	αIIbβ3, fibrinogen, IgG-FcγRIIa, thrombospondin, PAF receptor	84 211 212 213
*Streptococcus pyogenes*	M protein	αIIbβ3, fibrinogen, IgG-FcγRIIa	201 214
*Streptococcus sanguis*	SrpA	αIIbβ3, fibrinogen, IgG-FcγRIIa, GPIb	84 215 216 217

Abbreviations: Clf, clumping factor; FnBPA, fibronectin-binding protein A; IsdB, iron-regulated surface determinant B; PadA, platelet adherence protein A; PavB, pneumococcal adherence and virulence factor B; PspC, pneumococcal surface protein C; Sdr, serine-aspartate repeat protein; SpA, staphylococcal protein A; SrpA, serine-rich protein A; Ssp, stringent starvation protein; vWF, von Willebrand factor.

Platelets also express C–C motif and C–X–C motif chemokine receptors such as CCR1, CCR2, CCR4, and CXCR4, 86 which can detect all four classes of chemokines (C, CC, CXC, and CX _3_ C). These receptors allow platelets to recognize and prioritize chemotactic signals and result in rapid vectoring of platelets to sites of infection. 9 They are also involved in stimulating platelet adhesion, aggregation, and secretion. 87 Additionally, platelet activation leads to activation of the complement system, 88 89 and platelets also express various complement receptors after activation such as CR2, CR3, CR4, C3aR, C5aR, cC1qR, and gC1qR. 3 These may therefore serve as potential receptors for bacteria coated with complement factors, and lead to platelet aggregation. 11 Furthermore, an important class of receptors for pathogen sensing are TLRs, and platelets express numerous TLRs to detect the molecular features of microbes. 21 90 91 92 Platelets express, among others, functional TLR4, 93 as well as the accessory component for LPS signaling, including CD14, MD2, and myeloid differentiation primary response (MyD)-88. 94

### Bacterial Products Affect Platelet Functions

Platelets are able to respond to many bacterial products, and these products modulate platelet function. 25 LPS can stimulate platelet secretion of dense and α-granules through TLR4/MyD88 and cyclic guanosine monophosphate (cGMP)/cGMP-dependent protein kinase (PKG) signaling pathways. 94 This potentiates secretion-dependent integrin activation and platelet aggregation. Further to this, platelets recognize and discriminate between various isoforms of bacterial LPS and secrete differential cytokine profiles against these danger signals. 95 96 LPS also induces sCD40L release from platelets 97 as well as ROS generation. 98 Some sources of LPS can activate TLR2, 99 100 101 and this has also been implicated in LPS-induced cGMP elevation and platelet activation. 94 However, LPS is described as not always generating conventional platelet activation (e.g., typical P-selectin release from α-granules). 25 Bacterial structures from gram-positive bacteria such as lipoproteins, peptidoglycan, and LTA are TLR2 ligands, and also trigger platelet activation. 92 102 TLR activation in platelets induces a thromboinflammatory response, including platelet aggregation, formation of platelet–leukocyte complexes, and ROS generation 103 as well as the elaboration of acute-phase reactants like TNF-α. 91 However, studies have shown mixed effects of TLR2 agonists and LTA on platelet aggregation. 104 105

Platelets can migrate toward the chemotactic signal of bacterial N-formyl peptide by their receptors for this peptide. 106 The gingipain proteases HRgpA and RgpB from *P. gingivalis* activate platelet protease-activated receptor (PAR)-1 and PAR4, leading to platelet aggregation. 107 108 *S* . *aureus* α-toxin also causes platelet activation and leads to enhanced prothrombinase activity on the platelet surface. 109 110 Staphylococcal superantigen-like (SSL)-5 from *S* . *aureus* additionally induces platelet activation via platelet receptors GPVI and GPIb, 111 112 whereas the Panton–Valentine leukocidin toxin leads to platelet activation via neutrophil secretion products from damaged neutrophils. 113

Another class of exotoxins from *S* . *aureus* , extracellular adherence protein (Eap) and extracellular fibrinogen-binding protein (Efb) fibrinogen-binding proteins, also interacts with platelets. On the one hand, Eap enhances αIIbβ3 integrin activation, granule secretion, and aggregation, 114 whereas Efb inhibits platelet activation and aggregation 115 116 and has powerful antiplatelet actions. 117 *Staphylococcus aureus* enterotoxin B similarly inhibits platelet aggregation. 118 LTA from *S* . *aureus* has also been reported to inhibit platelet activation through platelet-activating factor (PAF) receptor and raised cyclic adenosine monophosphate (cAMP), 119 as well as to inhibit platelet aggregation, 120 121 122 but may support platelet adhesion to *Staphylococcus epidermidis* . 123 Additional products released by *S* . *aureus* also have opposing functions on platelet aggregation. While staphylothrombin mediates fibrin formation that supports aggregation, 124 staphylokinase prevents aggregation by degrading fibrinogen. 125

Bacterial toxins can also cause platelet destruction. For example, α-toxin from *S* . *aureus* and α-hemolysin from *E. coli* 126 as well as peptidoglycan from *S. aureus* 127 can induce platelet apoptosis. Indeed, these pore-toxins stimulate disturbances in the platelet membrane and can be cytotoxic. 3 128 *Escherichia coli* Shiga toxin causes downregulation of platelet CD47 expression, which leads to enhanced platelet activation and phagocytosis of platelets by macrophages. 129 Toxins such as pneumolysin from *Streptococcus pneumoniae* 130 and α-toxin from *S* . *aureus* 131 can cause platelet lysis, whereas streptolysin O from *Streptococcus pyogenes* 132 and phospholipase C from *Clostridium perfringens* 133 induce the formation of platelet–leukocyte complexes.

### Platelets Mediate Antimicrobial Attack

A further function of platelets in bacterial infection is mediating antimicrobial attack. Platelets mediate some of their antimicrobial actions through the secretion of potent antimicrobial proteins from their α-granules. 8 35 Moreover, platelets rapidly form clusters around bacteria that have been captured by Kupffer cells in the liver sinusoids (specialized macrophages in the liver), encasing the bacterium and facilitating its destruction. 13 Further, sCD40L causes increased generation and release of reactive oxygen (e.g., superoxide) and nitrogen (e.g., nitric oxide) species by platelets, which assists in pathogen destruction. 134 135

Platelets are able to bind and endocytose/phagocytose bacteria through engulfing endosome-like vacuoles that are formed by membrane endocytosis and become the site of α-granule release for the granular proteins to access the pathogen. 136 137 A mechanism of internalizing bacteria via the open canalicular system has also been proposed 138 (compare with Boukour and Cramer 139 ). Nonetheless, the platelet FcγRIIa receptor can bind IgG complexes and allows platelets to clear these complexes from the circulation. 140 Internalization of IgG-coated particles results in platelet activation and the release of RANTES and sCD40L. 141 Platelets opsonized by IgG can be destroyed by Fc-mediated platelet phagocytosis, contributing to the clearance of IgG-containing complexes from the circulation. 142 143 More broadly, activated platelets expose phosphatidylserine, and neutrophils have been shown to phagocytose activated platelets in a clearance program involving phosphatidylserine and P-selectin. 144 145 146

## Platelet Interactions with Viruses

**Fig. 6:**
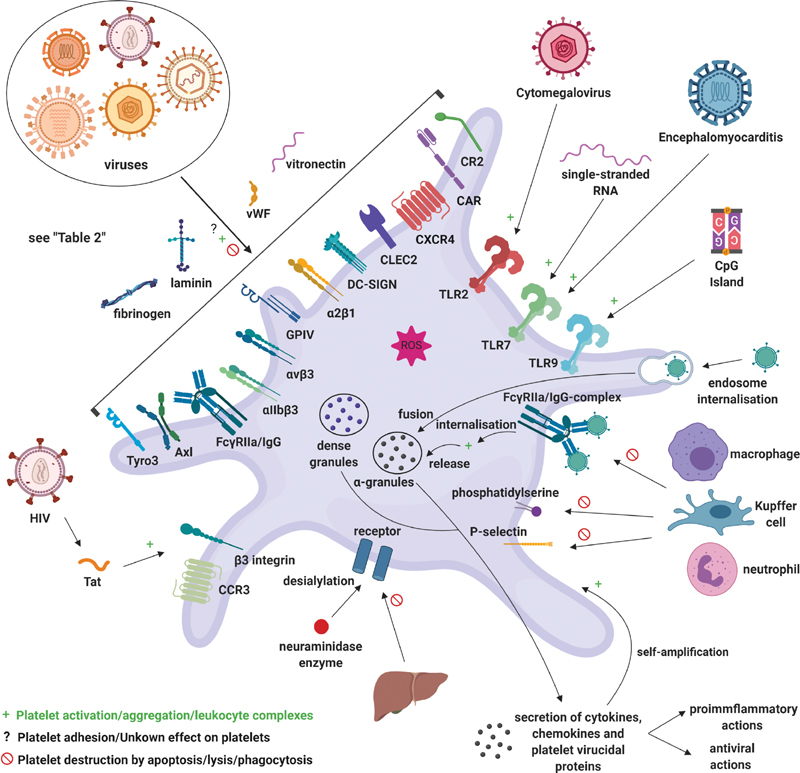
Platelet interactions with viruses. Various platelet receptors can mediate binding to viral particles; however, the direct effect of this binding on platelets is less well described than for bacteria. Pattern recognition receptors recognize classical viral signals, and viral products also modulate platelet function. Platelets mediate viral attack by secreting virucidal proteins and by engulfing viral particles, as well as by interacting with immune cells and enhancing the immune response. Overall, platelets may be activated and aggregate, but also face apoptosis. Virus–platelet aggregates and platelets with a viral load are targeted by leukocytes, and platelets are ultimately cleared from the circulation (created with https://biorender.com/ ). CAR, coxsackievirus and adenovirus receptor; CCR/CXCR, chemokine receptor; CLEC, C-type lectin-like receptor; CR, complement receptor; DC-SIGN, dendritic cell-specific ICAM-grabbing nonintegrin; FcγRIIa, low-affinity immunoglobulin gamma Fc region receptor II-a; HIV, human immunodeficiency virus; Ig, immunoglobulin; RNA, ribonucleic acid; ROS, reactive oxygen species; Tat, trans-activator of transcription; TLR, toll-like receptor; vWF, von Willebrand factor.

Viruses have been observed to interact directly with platelets. Various viruses have been identified adsorbed to or inside platelets, including influenza virus, 147 148 HIV, 136 149 150 hepatitis C, 151 152 153 and herpes simplex virus 154 as well as others such as vaccinia virus 155 and dengue virus. 156 157 158 However, the interactions between viruses and platelets are less well characterized compared with those of gram-positive bacteria. This section describes the interaction of platelets with viruses, which are summarized in Fig. 6 .

### Platelet Receptors in Viral Pathogen Sensing

Several platelet receptors have been identified to mediate binding to viral particles, 6 7 30 159 and are summarized in Table 3 . Similarly to bacteria, IgG is important for the adhesion of viral particles to platelets, where IgG-coated particles can interact with the FcγRIIa receptor 151 160 161 162 to be internalized into the platelet. 140 However, other antibody-dependent mechanisms that enhance viral binding to platelets are also described, 156 and platelets can further bind viruses in a receptor-independent manner. 163 For example, although the coxsackievirus and adenovirus receptor (CAR) is expressed on platelets, coxsackie B virus interaction with platelets has also been described independently of CAR and can result in P-selectin and phosphatidylserine exposure. 163 More broadly, β3 integrins are important platelet-adhesion receptors, and these receptors appear to facilitate viral adhesion to platelets. 18 65 164 Even though various receptors that are expressed on platelets have been implicated in viral adhesion and cell entry, the direct effect of this interaction on the platelet has not always been described.

**Table 3:** Platelet receptors that mediate viral binding

Virus	Viral component	Platelet receptors/host factors	Effect on platelet	References
Adenoviruses	Penton base (RGD ligand site)	Fibrinogen, laminin, vitronectin and vWF, αIIβ3, αvβ3, CAR receptor	Platelet activation, platelet–leukocyte aggregate formation	30 218 219 220 221
Dengue virus		DC-SIGN	Platelet activation, platelet apoptosis	178 222 223
Ebola virus		DC-SIGN		224
Enterovirus echovirus 9 strain Barty	VP1 capsid protein (RGD ligand site)	αvβ3		225
Epstein–Barr virus		CR2	Platelet activation	226
Hantaviruses		αIIβ3, αvβ3		227
Hepatitis C virus		GPVI		228
HIV	Mannose-type carbohydrates	CXCR4, DC-SIGN, CLEC2		174 229 230
Herpes simplex virus-1		αvβ3		231
Human parechovirus-1	VP1 capsid protein (RGD ligand site)	αvβ3		232
Lassa virus		DC-SIGN, Axl, Tyro3		233
Rotavirus	Spike protein VP4 (DGE ligand site)	α2β1		234 235

Abbreviations: CLEC2, C-type lectin-like receptor 2; DC-SIGN, dendritic cell-specific ICAM-grabbing nonintegrin; DGE, Asp-Gly-Glu tripeptide; HIV, human immunodeficiency virus; RGD, Arg-Gly-Asp tripeptide; VP, viral (capsid) protein; vWF, von Willebrand factor.

Platelets can also detect viruses through TLRs. Platelet TLR2 can bind cytomegalovirus, which triggers platelet activation, degranulation, and the formation of platelet–leukocyte aggregates. 165 TLR7 recognizes the classical viral PAMP, single-stranded RNA. 92 Platelets express functional TLR7, and activation via TLR7 leads to expression of CD40L and P-selectin, and P-selectin supports the adhesion of virally activated platelets to neutrophils. 22 166 Moreover, platelet TLR7 mediates complement C3 release from platelets, which in turn leads to platelet–neutrophil aggregation and NET release by neutrophils. 167 Encephalomyocarditis virus has been shown to interact with platelet TLR7. 166 Platelet TLR9 recognizes unmethylated CpG islands found in bacterial and viral DNA, which also leads to P-selectin surface expression. 92 168

### Viral Products Affect Platelet Functions

Viruses secrete various products that modulate platelet function. The secreted HIV Tat protein directly interacts with platelets in a process requiring the platelet receptors CCR3 and β3 integrin as well as calcium influx. This leads to platelet activation and CD40L expression as well as microparticle formation. 65 Indeed, platelet activation persists even in virologically suppressed HIV infection. 169 Viral enzymes such as neuraminidase can cause desialylation of platelet surface receptors, 6 and desialylation might promote platelet clearance in the liver. 170 171

### Platelets Mediate Antiviral Attack

The secretory products of platelets can also exert virucidal effects, including the inactivation of adenovirus, poliovirus and vaccinia virus, 172 and HIV suppression. 20 Moreover, platelets exhibit phagocytic behavior toward viruses such as HIV and can form engulfing vacuoles that lead to granular components being secreted on the virus particle, as described for bacteria. 136 Indeed, intact HIV-1 particles enclosed in endocytic vesicles have been found in the open canalicular system. 173 174 Recently, it has been proposed that platelets may also potentially phagocytose influenza virus. 175 176 Platelets may then cause disruption of viral integrity. 174 Overall, it has been suggested that internalization of viral particles by platelets may function to clear viruses from the circulation. 177

Viruses can cause the expression of P-selectin and phosphatidylserine exposure on platelets, and these components promote interactions with leukocytes as well as lead to phagocytosis of the platelet. 163 178 Interaction between platelets and viruses can also lead to sequestration to the reticuloendothelial system of the liver, where virus–platelet aggregates can be taken up by Kupffer cells and degraded. 179 Spleen macrophages also assist in clearing platelets with a viral load. 30

## Conclusion

Platelets are among the first cells to accumulate at sites of infection and inflammation, and can be considered as first responders to invading pathogens. Here, platelets have a key role in sensing and effecting the first wave of responses to microbial and viral threat. 8 9 This is achieved by the inflammatory activity of platelets but also through direct antibacterial and antiviral actions that facilitate the clearance of pathogens from the circulation. Platelets are therefore represented at the interface of hemostasis, inflammation, and antimicrobial host defense. Their position at the crossroads of these processes emphasizes their role as signaling entities in infection and inflammation.

**Fig. 7:**
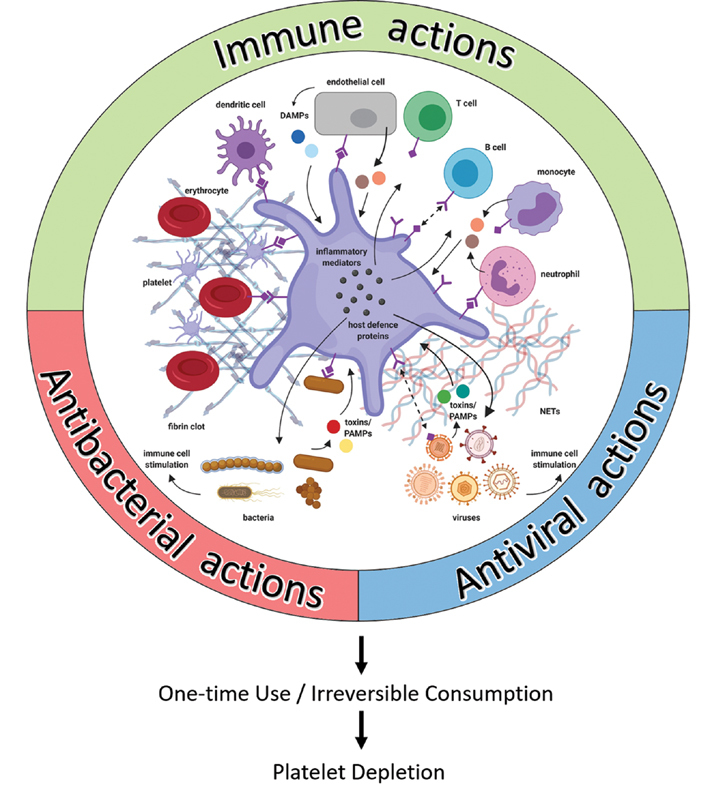
A generic large-scale cause for platelet dysfunction and depletion in infection. Platelets are intimately involved in the immune and host defense response to infection, where various stimuli challenge the platelet. Platelets operate in close connection with other cells and processes. Platelets are cells of one-time use, and their involvement in the diverse and interconnected processes against infection leads to their irreversible consumption. In the context of abundant stimulation, inappropriate and excessive activation of platelets results in their expenditure and exhaustion (created with https://biorender.com/ ). (Adapted from Yeaman 9 .) DAMP, damage-associated molecular pattern; NET, neutrophil extracellular trap; PAMP, pathogen-associated molecular pattern.

Various stimuli that are relevant to infection impinge on platelets, activating and forcing them to exert their effector actions. Recursive stimulation of activation receptors and successive activation of bystander platelets intensify the host-defense functions of platelets even at threshold stoichiometric ratios of platelets to pathogens. 180 Platelets face inappropriate activation and immunological destruction, and are inevitably consumed by their participation in host defense. An inflammatory milieu can thereby drive platelet dysfunction. In this review, we emphasize that platelet dysfunction can arise as a general consequence of an exaggerated systemic (immune) response to infection. Increased platelet consumption and removal can lead to thrombocytopenia, which is frequently observed during infection. Fig. 7 summarizes and links together the various processes we have discussed, to show a general mechanism of platelet depletion during infection.

Because of their largely protective role, lower platelet counts are associated with worse prognosis and greater likelihood of infection; however, platelets are also presented as having an ambivalent role in infections by possibly sheltering pathogens in certain cases. 6 7 9 12 30 181 Nonetheless, in the context of impairment of the immune system, the functions of platelets become more important. Following the contribution of platelets to diverse immunological processes, dysregulation of platelet–leukocyte interactions, which are important for inflammatory and immune reactions, together with dysregulation of inflammatory mediators, establish an excessive and unbalanced systemic inflammatory response. In this context, platelets can contribute to pathophysiological processes and immunopathology, and become dysfunctional.

Achieving a balance between pro- and anti-inflammatory responses during infection is difficult to manipulate effectively in a therapeutic context. Following from the diverse functions of platelets in infections, platelets are also placed at an interface between health and disease. Platelets are acutely affected by the surrounding environment. This, together with other characteristics of platelets such as their fast turnover, might position platelets as relevant signaling entities with clinical potential in disease tracking and targeting to evaluate or manage the course of infections. Although platelets are perhaps a lesser-known participant in the host-defense system, their large-scale depletion may cause significant health issues. Managing a generic depletion of platelets during the presence of infection should possibly be a more actively pursued clinical goal. The key points encapsulating the main ideas of this review are presented in Table 4 .

**Table 4:** Key points

• Platelets are versatile cells positioned at the interface of hemostasis, inflammation, and antimicrobial host defense, and their immune, antibacterial, and antiviral actions establish them as active participants in infection. • By nature of their normal functioning, platelets are invariably and irreversibly expended in the processes to which they contribute. • During infection, an onslaught of inflammatory and pathogen-derived stimuli can evoke and challenge platelets, leading to inappropriate activation, immunological destruction, and sequestration. • In the context of a dysregulated host response to infection, platelets can experience overwhelming activation and, consequently, consumption, and this represents a generic large-scale mechanism for platelet depletion in infection.
